# Proteção Miocárdica na Cirurgia Cardíaca – Qual o Método Ideal?

**DOI:** 10.36660/abc.20200622

**Published:** 2020-08-19

**Authors:** Ahmad Ali Abdouni

**Affiliations:** (1) Faculdade de Medicina Universidade de São Paulo São Paulo SP Brasil Faculdade de Medicina da Universidade de São Paulo, São Paulo, SP – Brasil

**Keywords:** Cirurgia Torácica, Revascularização Miocárdica, Ponte de Artéria Coronária, Cuidados Intraoperatórios, Soluções Cardioplégicas

O estudo da proteção miocárdica tem evoluído junto com a cirurgia cardíaca, no sentido de prevenir lesão miocárdica intraoperatória, que pode cursar com disfunção ventricular, arritmias, baixo débito cardíaco e outras complicações, muitas vezes irreversíveis.

Desde as primeiras cirurgias cardíacas com circulação extracorpórea (CEC) em 1953 com Dr. Gibbon no Massachusetts General Hospital,^1^ têm se estudado diversos métodos de proteção miocárdica, que permitissem cirurgias mais extensas do coração, com maior tempo de pinçamento da aorta. Nas primeiras cirurgias, a hipotermia vinha sendo o método de proteção miocárdica, mas mostrou-se insuficiente para tempos mais longos de isquemia.

Em 1955, Melrose et al.,^2^ publicaram no Lancet uma comunicação preliminar intitulada “Parada Cardíaca Eletiva”. O interessante é que a técnica de Melrose empregava sangue como veículo do citrato de potássio – lançando, portanto, as bases da proteção miocárdica com cardioplegia sanguínea, utilizando o potássio como solução despolarizante, nos moldes do que é realizado até os dias de hoje. Outros agentes foram utilizados posteriormente como magnésio, procaína, quelantes e bloqueadores de cálcio, eventualmente associados entre si, com a utilização de hipotermia ou não. Substratos, como glicose e oxigênio, podem ser fornecidos durante o período de clampeamento aórtico, para garantir algum metabolismo aeróbio nesse período. A adição de outros substratos, como glutamato, aspartato e lactato, assim como ATP ou creatina fosfato, precursores de intermediários do ciclo de Krebs, podem melhorar muito a proteção miocárdica. Diversos autores como Buckberg^3^ e, no nosso meio, Dr. Braile,^4^ se dedicaram a estudar diversas possibilidades para otimizar a proteção miocárdica.

Segundo diversos autores, o agente cardioplégico ideal necessita cumprir os seguintes requisitos:^5^

1. Parada cardíaca: indução rápida e eficaz da parada cardíaca com o miocárdio relaxado e com o mínimo de consumo de ATP;
2. Proteção miocárdica: efeitos protetores para retardar a lesão celular irreversível causada pela isquemia global e limitar a extensão da lesão de reperfusão;
3. Reversibilidade: reversão imediata da parada cardíaca com frequência cardíaca e força de contração, possibilitando “desmame” precoce da CEC;
4. Baixa toxicidade: meia-vida curta sem efeitos tóxicos em outros sistemas ou aparelhos após a descontinuidade da CEC.

Desde a sua introdução, as soluções cardioplégicas hipercalêmicas se tornaram o padrão ouro na preservação do miocárdio. A parada eletromecânica do coração pode ser atingida através da despolarização do potencial de membrana extracelular, o que reduz o potencial de repouso dos miócitos ventriculares. Após uma dose inicial, quando a quiescência eletromecânica é atingida, doses intermitentes (a cada 20 ou 30 minutos) são necessárias para manter a parada e evitar a disfunção miocárdica. A associação dessas soluções com o sangue do próprio paciente (cardioplegias sanguíneas) tem demonstrado redução nas dosagens das enzimas cardíacas e marcadores de reperfusão.^3^

A solução HTK (Custodiol ®) é uma solução cristaloide com baixa concentração de sódio e cálcio, que atua causando a hiperpolarização da membrana plasmática do miócito pela depleção do sódio no espaço extracelular, induzindo parada cardíaca na diástole (ao contrário da cardioplegia convencional – despolarizante). A combinação de histidina, triptofano e cetoglutarato na fórmula reduz a acidose intracelular, melhora a produção de ATP e estabiliza a membrana, reduzindo os danos da isquemia. A solução HTK tem sido aclamada como proteção ideal das cirurgias cardíacas de longa duração, sendo utilizada de rotina no transplante cardíaco, cirurgias pediátricas complexas, aneurismas de aorta e reoperações valvares na maioria dos centros, por oferecer proteção miocárdica por até 3 horas com infusão única da solução.^6^ Entretanto, esta solução requer cuidados específicos, como o manuseio da hipervolemia (são 20 – 25 ml/kg de solução infundidos logo após o pinçamento da aorta) e da acidose metabólica, comuns com a utilização desta solução.

A discussão sobre a utilização da solução HTK em cirurgias de revascularização do miocárdio é relevante, uma vez que ainda é a cirurgia cardíaca mais comum no Brasil. O trabalho de Gatti et al.,^7^ tem o mérito de demonstrar a não superioridade do Custodiol® em relação à solução cardioplégica convencional, o que é significativo no nosso meio, uma vez que a utilização desta solução implica em um custo adicional sem benefícios nesta população. Mesmo considerando somente casos mais complexos, com múltiplas anastomoses e tempo de pinçamento da aorta superior a 120 minutos, houve maior mortalidade no grupo que utilizou o Custodiol® (5,3% x 1,8%), relacionada a outros desfechos analisados como maior incidência de disfunção renal, transfusão sanguínea, tempo de internação em terapia intensiva e hospitalar, ainda que a diferença não tenha sido significativa na análise estatística. Na análise das variáveis relacionadas ao miocárdio, as duas soluções se mostraram equivalentes, sem diferença nas taxas de infarto, de baixo débito cardíaco, arritmia, de utilização de inotrópicos ou nos marcadores de necrose miocárdica (troponina). Alguns trabalhos têm sugerido a análise da função septal ventricular como o melhor marcador para determinar lesão miocárdica pós-operatória, uma vez que o septo interventricular corresponde a 35-40% da massa muscular ventricular total e é responsável por 80% da função ventricular direita.^8^ Reynolds^9^ reportou que 40% dos pacientes submetidos a cirurgia de revascularização do miocárdio e 60% dos pacientes com cirurgia valvar apresentam movimentação paradoxal do septo interventricular no período pós-operatório, o que denota algum grau de dano septal, muitas vezes transitório, sendo uma informação valiosa que deve ser obtida pelo ecocardiograma.

Sem dúvida, o Custodiol® tem seu papel em situações específicas, onde a utilização de dose única é tecnicamente importante como nos transplantes cardíacos, cirurgias pediátricas complexas e nas cirurgias minimamente invasivas, onde a infusão de doses repetidas de solução cardioplégica não é possível ou é tecnicamente mais difícil e sujeita a complicações.

Outras soluções alternativas continuam sendo estudadas para a proteção miocárdica. Em 1995, uma nova solução cardioplégica foi introduzida para cirurgias cardíacas congênitas, em um trabalho conduzido na Universidade de Pittsburgh.^10^ A solução, patenteada como “Solução de Del Nido”, é uma solução despolarizante mista de cardioplegia sanguínea e cristaloide que oferece proteção miocárdica segura por até 90 minutos com uma única dose. Além de conter cloreto de potássio como agente despolarizante, a fórmula contém ainda sulfato de magnésio, manitol, bicarbonato de sódio e lidocaína, e passou a ser utilizada também em cirurgias cardíacas de adultos de longa duração com excelentes resultados e com baixo custo, sendo uma alternativa às soluções comercialmente disponíveis.

Não existe a melhor técnica de proteção miocárdica. Apesar da grande variedade de soluções cardioplégicas disponíveis comercialmente, não existe um claro consenso sobre a composição ideal e as técnicas de utilização destas soluções. Além da escolha da solução para cada paciente, existe a questão da via de aplicação (anterógrada, retrógrada ou combinada), em dose única ou intermitente, associada ou não com hipotermia e outras possibilidades. Pinçamento (isquemia) intermitente sem a utilização de soluções cardioplégicas é outra técnica de proteção miocárdica utilizada por alguns grupos com bons resultados. O cirurgião deve estar apto a individualizar sua escolha e optar pela solução ou técnica mais adequada para cada paciente de acordo com seu planejamento cirúrgico, mas o custo também deve ser um fator a ser considerado no nosso meio, principalmente no Sistema Único de Saúde. Soluções mais dispendiosas devem ser reservadas para situações específicas com comprovado benefício.
