# Supplementary material for: Proteção Miocárdica na Cirurgia Cardíaca – Qual o Método Ideal?
Source: Arq Bras Cardiol. 2020 Aug 19;115(2):251–2. [Article in Portuguese] doi: 10.36660/abc.20200622 (PMC8384278; doi:10.36660/abc.20200622)
Supplement: Translation

# Translation: Myocardial Protection in Cardiac Surgery - What is the Ideal Method?

**Authors:** Ahmad Ali Abdouni

**Affiliations:** (1) Brazil Faculdade de Medicina da Universidade de São Paulo, São Paulo, SP – Brazil

**Keywords:** Thoracic Surgery, Myocardial Revascularization, Coronary Artery Bypass, Intraoperative Care, Cardioplegic Solutions

The study of myocardial protection has improved together with cardiac surgery, aiming to prevent intraoperative myocardial injury, which can lead to ventricular dysfunction, arrhythmias, low cardiac output and other complications, often irreversible ones.

Since the first cardiac surgeries with cardiopulmonary bypass (CPB) in 1953 performed by Dr. Gibbon at Massachusetts General Hospital,^1^ several methods of myocardial protection have been studied, allowing more extensive cardiac surgeries to be performed, with longer aortic clamping time. In the first surgeries, hypothermia was the method used for myocardial protection, but it showed to be insufficient for longer periods of ischemia.

In 1955, Melrose et al.^2^ published in the Lancet a preliminary communication entitled “Elective Cardiac Arrest”. The interesting thing is that the Melrose technique used blood as a vehicle for potassium citrate – thus laying the foundations for myocardial protection with blood cardioplegia, using potassium as a depolarizing solution, along the lines of what is done even today. Other agents were subsequently used, such as magnesium, procaine, chelators and calcium blockers, eventually associated among them, with the use of hypothermia or not. Substrates, such as glucose and oxygen, can be provided during the aortic clamping period, to ensure some aerobic metabolism during this period. The addition of other substrates, such as glutamate, aspartate and lactate, as well as ATP or creatine phosphate, precursors to Krebs cycle intermediates, can greatly improve myocardial protection. Several authors such as Buckberg^3^ and, in our country, Dr. Braile,^4^ have dedicated themselves to studying several possibilities to optimize myocardial protection.

According to several authors, the ideal cardioplegic agent must meet the following requirements:^5^

1. Cardiac arrest: rapid and effective induction of cardiac arrest with a relaxed myocardium and minimum ATP consumption;
2. Myocardial protection: protective effects to delay the irreversible cell damage caused by global ischemia and limit the extent of reperfusion injury;
3. Reversibility: immediate reversal of cardiac arrest with heart rate and contraction force, allowing an early “weaning” from CPB;
4. Low toxicity: short half-life without toxic effects on other systems or apparatus after CPB withdrawal.

Since its introduction, hyperkalemic cardioplegic solutions have become the gold standard in myocardial preservation. The electromechanical arrest of the heart can be achieved by depolarizing the extracellular membrane potential, which reduces the resting potential of ventricular myocytes. After an initial dose, when the electromechanical quiescence is achieved, intermittent doses (every 20 or 30 minutes) are necessary to maintain cardiac arrest and prevent myocardial dysfunction. The association of these solutions with the patient’s own blood (blood cardioplegia) has shown a reduction in the levels of cardiac enzymes and reperfusion markers.^3^

The HTK solution (Custodiol^®^) is a crystalloid solution with a low concentration of sodium and calcium, which acts by causing the hyperpolarization of the myocyte plasma membrane through the depletion of sodium in the extracellular space, inducing cardiac arrest in diastole (unlike conventional cardioplegia – depolarizing). The combination of histidine, tryptophan and ketoglutarate in the formula reduces intracellular acidosis, improves ATP production and stabilizes the membrane, reducing ischemic damage. The HTK solution has been praised as the ideal protection against long-term cardiac surgery, being routinely used in heart transplantation, complex pediatric surgeries, aortic aneurysms and valve reoperations in most centers, as it offers myocardial protection for up to 3 hours with a single infusion of the solution.^6^ However, this solution requires specific precautions, such as the management of hypervolemia (20 - 25 mL/kg of the solution infused right after the aortic clamping) and metabolic acidosis, which are usual when using this solution. The discussion about the use of the HTK solution in coronary artery bypass surgery is a relevant one, as it remains the most commonly performed cardiac surgery in Brazil. The work of Gatti et al.^7^ has the merit of demonstrating the non-superiority of Custodiol^®^in relation to the conventional cardioplegic solution, which is significant in our country, since the use of this solution implies in additional costs without benefits in this population.

Even when considering only more complex cases, with multiple anastomoses and aortic clamping time longer than 120 minutes, there was a higher mortality rate in the group that used Custodiol^®^(5.3% x 1.8%) which was related to other outcomes analyzed, such as greater incidence of renal dysfunction, blood transfusion, intensive care and hospital length of stay, although the difference was not significant in the statistical analysis. In the analysis of variables related to the myocardium, the two solutions were shown to be equivalent, with no differences in the rates of infarction, low cardiac output, arrhythmia, inotropic use or in the levels of myocardial necrosis markers (troponin). Some studies have suggested the analysis of ventricular septal function as the best marker to determine postoperative myocardial injury, as the interventricular septum corresponds to 35-40% of the total ventricular muscle mass and is responsible for 80% of the right ventricular function.^8^ Reynolds^9^ reported that 40% of patients submitted to myocardial revascularization surgery and 60% of patients with valve surgery have paradoxical movement of the interventricular septum in the postoperative period, which denotes some degree of septal damage, often transient, and that is a valuable information that must be obtained by echocardiogram.

Undeniably, Custodiol^®^has its role in specific situations, where the use of a single dose is technically important, such as in heart transplantations, complex pediatric surgeries and in minimally invasive surgeries, where the infusion of repeated doses of the cardioplegic solution is not possible or is technically more difficult and prone to complications.

Other alternative solutions are still being studied for myocardial protection. In 1995, a new cardioplegic solution was introduced for congenital heart surgery, in a study carried out at the University of Pittsburgh.^10^ The solution, patented as “Del Nido’s solution”, is a mixed depolarizing blood cardioplegia and crystalloid solution that offers safe myocardial protection for up to 90 minutes with a single dose. In addition to containing potassium chloride as a depolarizing agent, the formula also contains magnesium sulphate, mannitol, sodium bicarbonate and lidocaine, and is also being used in long-term cardiac surgeries in adults with excellent results and at low cost, being an alternative to commercially available solutions.

There is no best myocardial protection technique. Despite the wide variety of commercially-available cardioplegic solutions, there is no clear consensus about the ideal composition and techniques for using these solutions. In addition to choosing a solution for each patient, there is the question of the administration route (anterograde, retrograde or combined), in a single or intermittent dose, associated or not with hypothermia and other possibilities. Intermittent clamping (ischemia) without the use of cardioplegic solutions is another myocardial protection technique used by some groups with good results. The surgeons must be able to personalize their choices and choose the most adequate solution or technique for each patient according to the surgical planning, but the cost must also be a factor to be considered in our country, especially in the Public Unified Health System. More expensive solutions should be reserved for specific situations with proven benefit.
